# Polish Adaptation and Validation of the Intuitive (IES-2) and Mindful (MES) Eating Scales—The Relationship of the Concepts with Healthy and Unhealthy Food Intake (a Cross-Sectional Study)

**DOI:** 10.3390/nu14051109

**Published:** 2022-03-06

**Authors:** Aleksandra Małachowska, Marzena Jeżewska-Zychowicz

**Affiliations:** Department of Food Market and Consumer Research, Institute of Human Nutrition Sciences, Warsaw University of Life Sciences (SGGW-WULS), Nowoursynowska 159C, 02-776 Warsaw, Poland; marzena_jezewska_zychowicz@sggw.edu.pl

**Keywords:** intuitive eating, mindful eating, adaptation, validation, IES-2, MES, food intake

## Abstract

Intuitive (IE) and mindful (ME) eating share internally focused eating, yet previous studies have shown that these concepts are not strongly correlated, which suggests that they might be differently related to food intake. The study aimed to adapt the original Intuitive (IES-2) and Mindful (MES) Eating Scales to the Polish language, to test their psychometric parameters and, further, to examine associations of IE and ME with an intake of selected food groups, i.e., healthy foods (fresh and processed vegetables, fresh fruit) and unhealthy foods (sweets, salty snacks). A cross-sectional study was conducted in 2020 in a group of 1000 Polish adults (500 women and 500 men) aged 18–65 (mean age = 41.3 ± 13.6 years). The factor structure was assessed with exploratory (EFA) and confirmatory (CFA) factor analysis as well as structural equation modeling (SEM). Measurement invariance across gender was assessed with multiple-group analysis. Internal consistency and discriminant validity of the two scales was tested. Spearman’s correlation coefficient was used to examine the correlation between IES-2 and MES subscales with food intake. A 4-factor, 16-item structure was confirmed for IES-2, while EFA and CFA revealed a 3-factor, 17-item structure of MES. Both scales demonstrated adequate internal consistency and discriminant validity. Full metric and partial scalar invariance were found for IES-2, while MES proved partial invariances. “Awareness” (MES) and “Body–Food Choice Congruence” (IES-2) positively correlated with intake of healthy foods and negatively with the intake of unhealthy ones. “Eating For Physical Rather Than Emotional Reasons” (IES-2) and “Act with awareness” (MES) favored lower intake of unhealthy foods, whereas “Unconditional Permission to Eat” and “Reliance on Hunger and Satiety Cues” (IES-2) showed an inverse relationship. A greater score in “Acceptance” (MES) was conducive to lower intake of all foods except sweets. The results confirmed that adapted versions of the IES-2 and MES are valid and reliable measures to assess IE and ME among Polish adults. Different IE and ME domains may similarly explain intake of healthy and unhealthy foods, yet within a single eating style, individual domains might have the opposite effect. Future studies should confirm our findings with the inclusion of mediating factors, such as other eating styles, childhood experiences, dieting, etc.

## 1. Introduction

Intuitive and mindful eating are the two most commonly listed non-diet practices focused on the elimination of eating in response to external cues [1]. With the growing popularity of alternative weight management methods and also among health practitioners [2,3], it is of interest which approach would be most beneficial for maintaining health [1,4,5].

Intuitive eating (IE) is described as a process integrating food, mind, and body, which involves rejection of diet mentality [6,7]. Ten major IE principles were developed in 1995 by Tribole and Resch [8], yet its key features for the scholars and scientific purposes were determined in 2006 [7] and then revised in 2013 [9]. Four main IE components are: (1) eating in response to internal cues and allowing to eat whatever is desired (unconditional permission to eat), (2) eating only to satisfy physical hunger and not using food to cope with unpleasant emotions (eating for physical reasons), (3) determining when and how much to eat based on listening to hunger and satiety signals while eating (reliance on hunger and satiety cues), and (4) choosing foods that positively affect health as well as satisfy one’s sensory needs (body–food choice congruence) [7,9].

The use of mindfulness technique in the context of eating was firstly proposed by Kristeller and Hallet, who adapted it to create the Mindfulness-Based Eating Awareness Training (MB-EAT) in women suffering from binge eating disorder [10]. Even though there are no official definitions of mindful eating (ME), its major principles remain consistent in the literature [4]. ME is characterized by placing full awareness on the present moment of eating (e.g., avoiding external distractors such as watching television), paying attention on the effect of eating on all senses (i.e., sight, hearing, smell, taste, touch), and noticing all physical and psychological sensations and responses to certain foods (e.g., taste liking) but without the judgement [4,11].

While IE and ME seem to share a concept of internally focused eating and non-dieting approach [4], it is argued whether ME forms a part of IE or whether it gathers all IE principles with the addition of meditation [12]. There are still not enough studies assessing the correlation between IE and ME [11]. Anderson et al. [13] as well as Roman et al. [14] revealed that these concepts are not strongly correlated, whereas another study found an association among intuitive eating factors and mindfulness but not ME solely [15]. On the other hand, Kerin et al. [11] found that some IE and ME domains may be moderately correlated, while others do not reveal a similar effect.

The existing studies have shown that both IE and ME may be associated with better psychological health indicators, such as body and image acceptance, higher self-esteem, or lower frequency of disordered eating behaviors [4,12,16,17], but can also be beneficial for physiological parameters [5,12,16]. Nevertheless, the outcomes of the previous studies assessing how intuitive and mindful eating are linked with dietary habits are inconsistent, including positive, negative, or non-significant results [1,18,19,20,21,22,23,24,25,26,27]. Moreover, incoherent results on the association between IE and ME [11,13,15] might suggest that they can have different effects on food intake. Taking those issues into consideration, there is a need for further studies, which would simultaneously include IE, ME, and their link with food intake.

In the available research, intuitive eating was measured with the Intuitive Eating Scale [7] or its newest version improved by the authors of IES, the Intuitive Eating Scale-2 [9]. IES-2 incorporates an additional, fourth IE domain (“Body–Food Choice Congruence”), coherent with IE principles presented by the authors of this construct [8]. On the other hand, there are several measures to assess ME, for example, the Mindful Eating Questionnaire [28], the Mindful Eating Scale [29], the Expanded Mindful Eating Scale [30], the Mindful Eating Inventory [31], the Mindful Eating Behavior Scale [32], or the Four-Facet Mindful Eating Scale [33]. Nonetheless, to our knowledge, no adaptation study was conducted in the Polish representative population, which limits the possibility to use any of the mentioned instruments. However, few studies in Poland included the construct of mindful eating [34,35,36], which was assessed with the Mindful Eating Scale (MES) [29]. Factorial structure of MES was not tested in any of these studies, yet internal consistency measured with Cronbach’s alpha reliability coefficient revealed satisfactory values [34,35,36]. Moreover, to our knowledge, no studies on intuitive eating in the Polish population were published nor with the use of IES or IES-2; hence, the validity of these scales has not been previously tested in the Polish population.

The aim of the study was threefold: (1) to adapt Intuitive Eating Scale-2 (IES-2) and Mindful Eating Scale (MES) into Polish and to test their psychometric parameters, (2) to investigate the correlation between intuitive eating (IE) and mindful eating (ME), and (3) to assess association of IE and ME with the intake of selected food groups, i.e., vegetables (fresh and processed), fresh fruit, sweets, and salty snacks, in women and men. We hypothesize that the original structure of the IES-2 and MES would not be replicated due to the cultural differences, yet psychometric parameters of the obtained models will be satisfactory. Moreover, our hypothesis is that IE and ME domains would not be strongly correlated and that they will be differently correlated with food intake, including differences observed between a group of women and men.

## 2. Materials and Methods

### 2.1. Study Design and Sample Collection

Data were collected in February 2020 through a cross-sectional survey. The study sample was recruited by a professional market research agency from an e-panel consisting of around 60,000 registered individuals. CAWI (Computer-Assisted Web Interview) technique was used to collect survey data. To ensure sample representativeness, quota controls, such as age, place of residence, and region, were set. Only participants aged 18–65 years were included in the study. Participance in the study was voluntary. Informed consent was obtained from all the study participants before the study. The final study sample included 1000 participants (500 women and 500 men). Data confidentiality as well as anonymity were assured. The study was approved by the Ethics Committee of the Institute of Human Nutrition Sciences, Warsaw University of Life Sciences, in Poland (Resolution No. 02/2020).

### 2.2. Instruments: Intuitive Eating Scale-2 (IES-2) and Mindful Eating Scale (MES)

Intuitive eating was assessed with the Intuitive Eating Scale-2 (IES-2) [9]. This 23-item measure consists of 4 subscales: (1) Unconditional Permission to Eat (6 items, e.g., “If I am craving a certain food, I allow myself to have it.”); (2) Eating for Physical Rather Than Emotional Reasons (8 items, e.g., “I am able to cope with my negative emotions (e.g., anxiety, sadness) without turning to food for comfort.”); (3) Reliance on Hunger and Satiety Cues (6 items, e.g., “I rely on my hunger signals to tell me when to eat.”); and (4) Body–Food Choice Congruence (3 items, e.g., “I mostly eat foods that give my body energy and stamina.”). In the current study, original coding and scoring procedure was used. Participants were asked to rate each item from strongly disagree (1) to strongly agree (5). A mean score was calculated for each subscale (range 1–5), with higher scores reflecting higher level of intuitive eating.

The 28-item Mindful Eating Scale (MES) [29] was used to measure mindful eating. The MES consists of 6 subscales: (1) Acceptance (6 items, e.g., “I tell myself I shouldn’t be eating what I’m eating.”); (2) Awareness (5 items, e.g., “I notice how my food looks.”); (3) Non-reactivity (5 items, e.g., “I can tolerate being hungry for a while.”); (4) Routine (4 items, e.g., “I eat the same thing on the same day of each week.”); (5) Act with awareness (4 items, e.g., “I eat something without really being aware of it.”); and (6) Unstructured eating (4 items, e.g., “I eat between meals.”). Original coding and scoring procedure was used in the current study. Participants rated each item from rarely/never (1) to usually/always (4). A mean score was calculated for each subscale (range 1–5), with higher scores reflecting higher level of mindful eating.

Polish adaption of the IES-2 and MES was conducted by two independent translators. The process included several stages: initial translation into Polish, synthesis of the two versions, back-translation into English, corrections, preparation of the prefinal version, pretesting in a group of 49 students, and further agreement on the final version (Appendix A and Appendix B) [37].

### 2.3. Food Intake

A food frequency questionnaire (KomPAN^®^) [38] was used to measure the frequency of the consumption of the selected food groups in the last three months: vegetables (fresh and processed, separately), fresh fruit, sweets, and salty snacks. The frequency was evaluated in 6 following categories: never (1), 1–3 times a month (2), once a week (3), a few times a week (4), once a day (5), and a few times in a day (6). Those values were converted to reflect the daily frequency of intake, ranging from 0 to 2 times/day [39]. Moreover, participants declared how many portions of products they eat daily given that 1 portion of vegetables and fruit (both fresh and processed) equals 100 g, and 1 portion of sweets and salty snacks are 50 g. Exemplary portion sizes were added to each question. To obtain the final value reflecting the daily intake of each food group, the daily frequency of intake was multiplied by portions consumed per day. Daily food intake was categorized into terciles, with the 1st tercile indicating “low intake” and 3rd tercile “high intake”.

### 2.4. Statistical Analysis

Descriptive statistics were used to present sociodemographic characteristics. The diversity of socio-demographics between women and men was tested with the independence χ^2^ test.

Exploratory factor analysis (EFA) with varimax rotation with Kaiser normalization was conducted to confirm the factorial structure of the IES-2 and MES. Certain criteria were set to determine final factors: eigenvalue ≥ 1.0, a scree plot test, interpretability of the solution, and factor loadings of at least 0.50. Kaiser–Meyer–Olkin (KMO) measure of sampling adequacy and Bartlett’s test of sphericity confirmed data factorability [40].

The fit of the factorial structure identified during EFA was tested by running confirmatory factor analysis (CFA). The following model fit indices were assessed: the chi-square fit statistics/degree of freedom (χ^2^/df), Tucker–Lewis index (TLI), comparative fix index (CFI), root mean square error of approximation (RMSEA), and root mean square residual (RMR). Acceptable values of mentioned parameters were: χ^2^/df below 2 or 3; TLI ≥ 0.95; CFI ≥ 0.95; RMSEA < 0.06; and the smallest RMR possible [41]. Models were modified until they revealed satisfactory fit parameters by correlating error variances and eliminating items with low loading values and/or high standardized residual covariances (>4) with multiple items [42].

Configural, metric, and scalar invariance of the final models of IES-2 and MES were tested across gender [43]. The chi-square difference test was selected to examine changes in the fit among models. *p*-Value above 0.05 indicated non-significant changes [43]. If the *p*-value was below 0.05, items that were causing a significant decrease of the model fit were allowed to be freely estimated across gender groups to achieve partial measurement invariance [43].

The internal consistency of items within each identified factor was tested using Cronbach’s alpha, with values higher than 0.70 considered acceptable.

Mean factor scores for the IES-2 and MES in the total sample and separately in women and men were presented with descriptive statistics. Normality of the distribution was checked by conducting The Shapiro–Wilk test. The Mann–Whitney U Test was applied to compare differences between women and men in factor scores. *p*-Value lower than 0.05 was considered significant.

Spearman’s correlation was used to measure the association between intuitive and mindful eating components as well as their relationship with the intake of selected food group products.

Discriminant validity was assessed by running the Mann–Whitney U test. Mean scores for IES-2 and MES subscales were compared between two groups identified based on intake of favorable (fresh and processed vegetables and fresh fruit) and unfavorable (sweets and salty snacks) foods, i.e., low intake, 1st tercile; high intake, 3rd tercile.

The analyses were carried out using IBM SPSS Statistics for Windows, version 26.0 (IBM Corp, Armonk, NY, USA) and AMOS graphics version 27.0.

## 3. Results

### 3.1. Characteristics of the Study Sample

Sociodemographic characteristics of the study sample are presented in Table 1. The study group included 1000 individuals (equally numbered groups of women and men) aged 18–65 years, with a mean age of 41.3 years (±13.6 standard deviation (SD)). There were no significant differences among gender groups according to age, education, and place of residence.

### 3.2. Factor Structure of the IES-2

EFA revealed a four-factor structure of the IES-2. The total variance explained was 60.29%. Three items (Item 12, 13, and 15) had a factor loading below 0.5; thus, they were eliminated from further analysis (Table 2).

Confirmatory Factor Analysis (CFA) was used to test the fit of the model with four correlated latent variables identified during EFA (Table 3). As the initial model proved unsatisfactory parameters (Model a), several modifications were introduced. Firstly, Item 14 was eliminated due to the low loading value (0.66) (Model b). Then, based on modification indices, error variances of the items within the same factor were allowed to correlate (Items 8 and 23, 1 and 9, 1 and 4, 1 and 20, 9 and 19, 4 and 9, 19 and 20, 4 and 20; Model c). Item 4 was further removed due to the low loading value (0.67; Model d). Finally, Item 9 was eliminated, as it had high residual covariances (Model e).

The final model consisted of 16 items within four factors named as follows: Factor 1—"Reliance on Hunger and Satiety Cues” (RHSC, Items 6–8 and 21–23, Cronbach’s alpha = 0.888); Factor 2—"Eating For Physical Rather Than Emotional Reasons” (EPR, Items 2, 5, 10, and 11, Cronbach’s alpha = 0.859); Factor 3—"Body–Food Choice Congruence” (B-FCC, Items 1, 19, and 20, Cronbach’s alpha = 0.763); Factor 4—"Unconditional Permission to Eat” (UPE, Items 3, 16, and 17, Cronbach’s alpha = 0.747).

### 3.3. Multi-Group Analysis

Measurement invariance across gender was tested by comparing fit measures of created models (Table 4). Metric invariance was supported (*p* > 0.05), whereas scalar invariance was not confirmed (*p* < 0.05). Three items were found to significantly decrease model fit (Item 7, *p* = 0.020; Item 1, *p* < 0.001; and Item 16, *p* = 0.028); thus, they were allowed to be freely estimated by releasing the constraints across gender groups. As a result, partial scalar invariance was achieved (Adj. Model 2).

Mean scores for IES-2 subscales were calculated (Table 5). Based on EFA results, originally reverse-scored Item 1 turned out to have negative factor loading; thus, reverse-scoring of this item was no longer needed. Females were found to present a higher level of “Body–Food Choice Congruence”, whereas men displayed greater intensity of “Eating For Physical Rather Than Emotional Reasons”. Two remaining subscales did not significantly differ among women and men.

### 3.4. Factor Structure of the MES

EFA revealed a five-factor solution, which explains 53.27% of the total variance (Table 6). However, Factor 5 was eliminated from further analysis, as only one item (Item 9) loaded on that factor. Moreover, Item 28 was removed due to inadequate factor loading.

Confirmatory Factor Analysis (CFA) was used to test the fit of the model with 4 correlated latent variables (Table 7). The initial model proved unsatisfactory parameters (Model a); hence, several modifications were introduced. Item 6 and 23 were eliminated due to relatively low loading values (0.69 and 0.71, respectively; Model b). Secondly, based on modification indices, error variances of the items within the same factor were allowed to correlate (Items 1 and 7, Items 3 and 14, Items 3 and 21, Items 10 and 14, Items 10 and 17, Items 11 and 18, Items 14 and 17, Items 14 and 21, Items 16 and 19, Items 16 and 22; Model c). Finally, Items 3, 7, 12, 16, and 19 were removed because of high residual covariances, which resulted in satisfactory fit indices values (Model d). Internal consistency of items within factors have been checked with the following results of Cronbach’s alpha: Factor 1—0.874 (Items 5, 10, 11, 14, 17, 18, 21, 24, and 25), Factor 2—0.832 (Items 2, 8, 15, and 26), Factor 3—0.806 (Items 1, 7, 13, 20, and 27), and Factor 4—0.679 (Items 4 and 22). Unacceptable value for Factor 4 combined with the fact that it consisted of two items only prompted us to eliminate it and retest the model with three factors remaining (Model e). Lastly, to obtain the final model with χ^2^/df lower than 3, Item 7 with high standardized residual covariance values was removed (Model f).

The final model consisted of 17 items within three factors named as follows: Factor 1—"Act with awareness” (Items 5, 10, 11, 14, 17, 18, 21, 24, and 25, Cronbach’s alpha = 0.874); Factor 2—"Awareness” (Items 2, 8, 15, and 26, Cronbach’s alpha = 0.832); and Factor 3—"Acceptance” (Items 1, 13, 20, and 27, Cronbach’s alpha = 0.756).

### 3.5. Multi-Group Analysis

Fit measures of created models were compared to test measurement invariance across gender (Table 8). Metric non-invariance was found (*p* < 0.05); thus, the constraints for sources of non-invariance (Item 20, *p* = 0.002; Item 13, *p* = 0.014) were released, and the model was retested, resulting in partial metric invariance (Adj. Model 1). Scalar invariance was not confirmed either (*p* < 0.05); therefore, the constraints for two items (Item 11, *p* = 0.004; Item 18, *p* = 0.010), which were found to decrease model fit, were released, resulting in partial scalar invariance (Adj. Model 2).

Mean scores were calculated for each identified MES subscale (Table 9). Significant difference between women and men was noted only for “Awareness” subscale.

### 3.6. Relationship between Intuitive and Mindful Eating and Their Association with Food Intake

The relationship between IES-2 and MES factors was tested (Table 10). Moderate correlations were found between “Eating For Physical Rather Than Emotional Reasons” and two MES subscales—"Act with awareness” and “Acceptance” (r = 0.487 and r = 0.401, respectively).

The discriminant capability of IES-2 and MES was presented in Table 11. Participants characterized by high intake (3rd tercile) of fresh and processed vegetables and fresh fruit scored higher in “Awareness” and “Body–Food Choice Congruence” subscales. Those characterized by low intake of these products (1st tercile) scored higher in “Acceptance” and “Eating For Physical Rather Than Emotional Reasons”. Higher scores for “Act with awareness”, “Acceptance”, “Eating For Physical Rather Than Emotional Reasons”, and “Body–Food Choice Congruence” favored lower intake of sweets and salty snacks, whereas participants from the 3rd tercile of sweets and salty snacks intake scored higher in “Reliance on Hunger and Satiety Cues” and “Unconditional Permission to Eat”.

IES-2 and MES factors were correlated with the intake of selected food groups in grams (Table 12). Awa, similar to B-FCC, positively correlated with intake of both fresh and processed vegetables and fresh fruit in the total group as well as in women and men separately. Positive correlations were also observed for RHSC and intake of fresh fruit (except for men) as well as sweets, which were also found to correlate with UPE yet in a greater manner among women than in men. It was noted that the higher the score in UPE, the greater the intake of salty snacks in women. Negative correlations were found between ActAwa and EPR and intake of sweets and salty snacks. Awa also negatively correlated with intake of salty snacks in the total group and both women and men. The higher the Acc score, the lower was the intake of salty snacks and fresh vegetables in women and men and fresh fruit in men. A greater score in B-FCC was linked with a lower intake of sweets in women.

## 4. Discussion

The original version of the Intuitive Eating Scale-2 [9] was previously adapted and validated in diverse populations [14,44,45,46,47,48,49,50,51,52,53]. Most studies confirmed a four-factor structure of the IES-2 [44,45,47,48,49,50,51,53]; yet, it should be noted that women, especially young or middle-aged, constituted the majority of the study samples [44,45,47,49,50,51]. A few studies included a similar number of women and men within a broader age range [46,47,48,53], yet only two of them confirmed the original factorial structure of the IES-2 [48,53], similar to our results obtained from a representative study sample consisting of adults aged 18–65. Those incoherent results suggest that the IES-2 structure may vary in different settings and cannot be replicated in any sample without previous testing. All items within our final model loaded into the same factors as in the original IES-2 except Item 1, originally reverse-scored and loaded into UPE subscale, i.e., “I try to avoid certain foods high in fat, carbohydrates, or calories”. In the current study, it loaded into the B-FCC subscale, and reverse-scoring was revoked based on EFA results. B-FCC is described as an ability to match food choices with one’s body needs, i.e., eating mostly nutritious foods that increase energy levels, etc. [9]. Limiting intake of foods that worsen body functioning as a conscious, voluntary choice yet not in a form of imposed restrictions seems to be in line with B-FCC.

Despite being used in several studies [11,14,34,35,36], Mindful Eating Scale [29] was not previously adequately tested in different cultures, languages, and countries. In our study, the original six-factor MES structure was not confirmed, and several items have been removed, as they negatively affected model fit indices. Our results suggest that more studies with MES are needed to confirm its structure, including testing correlations between MES and other scales used to measure ME [28,30,31,32,33] to ensure that MES is a reliable and valid tool to assess ME.

According to the research, intuitive and mindful eating may be associated with a greater ability of food intake regulation [4,12,16,17]. However, the outcomes of the previous studies assessing how intuitive and mindful eating are linked with certain dietary habits are inconsistent, which confirms the need for further investigation [1,18,19,20,21,22,23,24,25,26,27]. Some studies suggest possible beneficial correlations between selected components of those eating styles and eating behaviors, including greater intake of favorable foods, such as fruit and vegetables, and a lower intake of energy-dense food [18,20,21,22,23,54]. This was partially confirmed by our results, which primarily concern the positive relationship between Awa (MES) and B-FCC (IES-2) scores and consumption of fresh and processed vegetables and fresh fruit. Making conscious food choices, for both men and women, including appearance, smell, and taste of food but also the adjustment of food intake to the body (its good functioning, energy, condition) accompanied by lower intake of products high in fat, carbohydrates, and calories, promotes consumption of favorable foods.

Although in previous studies, intuitive eating subscales (except for UPE) were generally associated with healthier food choices and greater diet quality [18,20,21], in our study, it was confirmed only for B-FCC and intake of fresh fruit and fresh and processed vegetables. In addition, a positive correlation between intake of fresh fruit and reliance on hunger and body signals (RHSC) was observed in the female group. On the other hand, lack of emotional eating (EPR) was associated with a lower intake of sweets and salty snacks in both men and women. Thus, the positive association with higher dietary quality was also confirmed.

As in other studies, a higher UPE score as well as RHSC was not associated with higher diet quality, as it correlated positively with the intake of sweets. Previous studies on intuitive eating have confirmed the relationship of UPE with a greater intake of sweets and salty snacks [18,20,21]. In our study, only women with a higher score of unconditional permission to eat showed a higher intake of salty snacks. Furthermore, a lower intake of fruit and vegetables was not confirmed [18,20,21], and even a weak positive correlation with fresh fruit consumption has been shown. The positive correlations between RHSC, UPE, and intake of sweets might show that intuitive eating, especially in the initial phase, can be challenging to implement, resulting in less favorable eating behaviors, as this approach rejects restrictions in quality and quantity of food [12]. RHSC is characterized by eating in response to internal hunger and satiety cues [7]. Despite being an innate ability, RHSC may be disrupted by childhood food experiences associated with parental feeding practices that cause children to consume more food than the body needs [55,56]. Dieting, disordered eating, or eating disorders appearing in the past can also alter the sensation of hunger and satiety [7,57]. For example, higher intake of unfavorable foods as a result of unconditional permission to eat what is desired (UPE) might be only a temporary effect observed among individuals previously engaging in rigid dietary control and restrictions, known as a risk factor for excessive consumption or eating for reasons unrelated to physical hunger [58,59]. Our results may be due to the influence of previous individuals’ experiences, yet such factors were not included in our study. It is suggested to incorporate them as mediators into future studies on the relationship between eating style, eating behavior, and food intake.

“Awareness” (Awa), which concerns physical sensations that occur while eating, such as food aroma, textures, smell, and how it looks [29], positively correlated with consumption of fresh and processed vegetables and fresh fruit. In contrast, greater ActAwa and Acc correlated with lower consumption of unfavorable foods (sweets and salty snacks), which may improve diet quality. Similar observations, especially in comparison with ActAwa, were noted for the EPR factor (IES-2). Both ActAwa and Acc were moderately positively correlated with EPR (IES-2), which may explain these findings. Nevertheless, Acc also correlated with lower consumption of fresh and processed vegetables. Thus, this element of mindful eating may contribute to a lower amount of food consumed in general.

Several differences among women and men were found, which proves that studies on IE and ME and their association with food intake should examine this relationship separately for those two groups. Among women, ActAwa and RHSC correlated positively with fresh fruit intake and UPE with salty snacks intake, whereas a negative correlation was found for B-FCC and sweets. On the other hand, in men, negative correlations were observed for fresh vegetables and EPR as well as for fresh fruit and Acc, while Awa was positively correlated with sweets intake. Significant differences between women and men in EPR, B-FCC, and Awa scores (Table 5 and Table 9) may partially explain some of our observations. Another factor that could have affected those results are differences in dietary intake among women and men, i.e., lower/greater intake of certain foods [60,61,62]. Additionally, the above-mentioned negative experiences from the past might have had mediating role [7,55,56,57].

According to the hypothesis, original structure of the IES-2 and MES was not replicated, which can both result from cultural differences and the use of representative study sample; nevertheless, final models proved satisfactory psychometric parameters. IE and ME domains were not strongly correlated, as expected, which may explain several differences observed in their relationship with food intake. As intended, correlations between IE and ME domains with food intake varied between women and men.

The current study as well as others conducted with the use of validated versions of IES-2 and MES will be useful for better understanding of the factors, such as feelings, food motives, and thoughts about food, in explaining food choices. In addition, their results will provide a better understanding of alternative weight control methods, which simultaneously positively influence food intake, i.e., higher consumption of favorable and lower consumption of unfavorable foods. Observations on gender differences in IE, ME, and food choices might be used in developing strategies addressed directly to women and men, aimed at improving diet quality, health, and food beliefs.

### Strengths and Limitations

Adaptation and validation of the IES-2 and MES were conducted in a sample representative for the Polish population, which can be pointed as a study strength. Moreover, this is the first study to assess simultaneously IE and ME and their correlation with food intake among Polish adults. Nevertheless, a few limitations should be mentioned. Convergent validity of the IES-2 and MES as well as their test-retest reliability were not tested in our study. Moreover, the causality of associations cannot be determined due to the cross-sectional design of the study. Correlations between IE, ME, and food intake were examined only with the use of selected food groups. As the study was conducted in February, and questions about food intake related to the last 3 months of the consumption, the aspect of seasonal changes in dietary intake was not included.

## 5. Conclusions

Our study supported a 4-factor, 16-item structure of the Intuitive Eating Scale-2 (IES-2), while a 3-factor, 17-item solution was revealed for the Mindful Eating Scale (MES). A valid Polish version of IES-2 and MES might be useful in further investigation of factors influencing eating behaviors in this population. Different IE and ME domains may similarly explain intake of healthy and unhealthy foods yet within a single eating style individual domains might have the opposite effect. Nonetheless, future studies should confirm our findings on IE, ME, and food intake relationships in women and me, also with the use of other scales to measure ME. It is also suggested that further research would include mediating the role of factors, such as other eating styles, childhood experiences, dieting, or eating disorder episodes, on the relationship between IE, ME, and healthy or unhealthy food intake.

## Figures and Tables

**Table 1:** Socio-demographic characteristics of the study sample.

Variables		Total (*N* = 1000) *N* (%)	Women (*N* = 500) *N* (%)	Men (*N* = 500) *N* (%)
**Age (in years)**	18–24	112 (11.2)	61 (12.2)	51 (10.2)
	25–39	351 (35.1)	188 (37.6)	163 (32.6)
	40–54	304 (30.4)	133 (26.6)	171 (34.2)
	55–65	233 (23.3)	118 (23.6)	115 (23.0)
**Education**	Primary	171 (17.1)	91 (18.2)	80 (16.0)
	Lower secondary	240 (24.0)	119 (23.8)	121 (24.2)
	Upper secondary	343 (34.3)	163 (32.6)	180 (36.0)
	Higher (e.g., BSc, MSc)	246 (24.6)	127 (25.4)	119 (23.8)
**Place of Residence**	Village	373 (37.3)	203 (40.6)	170 (34.0)
	Town below 20,000 inhabitants	131 (13.1)	62 (12.4)	69 (13.8)
	Town between 20,000 and 100,000 inhabitants	183 (18.3)	87 (17.4)	96 (19.2)
	City over 100,000 inhabitants	313 (31.3)	148 (29.6)	165 (33.0)

*N*, number of participants; BSc, Bachelor of Science; MSc, Master of Science.

**Table 2:** Component loadings for IES-2 items.

IES-2 Statements	Original Factor	Items	Factor 1	Factor 2	Factor 3	Factor 4
I trust my body to tell me how much to eat.	RHSC	8	0.820 *	−0.020	0.131	0.168
I trust my body to tell me when to stop eating.	RHSC	23	0.797 *	−0.064	0.086	0.206
I trust my body to tell me when to eat.	RHSC	6	0.715 *	0.021	0.191	0.313
I rely on my hunger signals to tell me when to eat.	RHSC	21	0.707 *	−0.059	0.182	0.227
I rely on my fullness (satiety) signals to tell me when to stop eating.	RHSC	22	0.704 *	0.001	0.221	0.223
I trust my body to tell me what to eat.	RHSC	7	0.703 *	−0.122	0.222	0.194
I find myself eating when I’m feeling emotional (e.g., anxious, depressed, sad) even when I’m not physically hungry. ^a^	EPR	2	−0.048	0.837 *	−0.073	0.017
I find myself eating when I am stressed out even when I’m not physically hungry. ^a^	EPR	11	−0.082	0.825 *	−0.086	−0.041
I find myself eating when I am lonely even when I’m not physically hungry. ^a^	EPR	5	−0.025	0.800 *	−0.100	−0.053
I use food to help me soothe my negative emotions. ^a^	EPR	10	−0.035	0.797 *	−0.141	0.032
I try to avoid certain foods high in fat, carbohydrates, or calories. ^a^	UPE	1	0.144	−0.137	−0.736 *	0.090
I have forbidden foods that I don’t allow myself to eat. ^a^	UPE	9	0.087	−0.196	−0.702 *	−0.123
I mostly eat foods that make my body perform efficiently (well).	B-FCC	19	0.411	−0.018	0.630 *	0.186
I get mad at myself for eating something unhealthy. ^a^	UPE	4	−0.010	−0.450	−0.602 *	−0.053
I mostly eat foods that give my body energy and stamina.	B-FCC	20	0.455	−0.050	0.583 *	0.151
Most of the time, I desire to eat nutritious foods.	B-FCC	18	0.264	−0.264	0.392	0.338
I allow myself to eat what food I desire at the moment.	UPE	16	0.354	−0.232	−0.102	0.670 *
I do NOT follow eating rules or dieting plans that dictate what, when, and/or how much to eat.	UPE	17	0.369	−0.027	−0.067	0.650 *
When I am lonely, I do NOT turn to food for comfort.	EPR	14	0.095	0.191	0.316	0.639 *
If I am craving a certain food, I allow myself to have it.	UPE	3	0.381	−0.245	−0.121	0.625 *
I am able to cope with my negative emotions (e.g., anxiety, sadness) without turning to food for comfort.	EPR	12	0.358	0.343	0.396	0.492
I find other ways to cope with stress and anxiety than by eating.	EPR	15	0.308	0.187	0.455	0.477
When I am bored, I do NOT eat just for something to do.	EPR	13	0.219	0.101	0.418	0.469
*Eigenvalue*			4.481	3.325	3.189	2.870
*% of Variance explained*			19.481	14.458	13.867	12.480
Cronbach’s Alpha **			0.888	0.859	0.777	0.712

^a^ reverse-coded items; ^b^ original IES-2 number of the statement; UPE, Unconditional Permission to Eat; EPR, Eating For Physical Rather Than Emotional Reasons; RHSC, Reliance on Hunger and Satiety Cues; B-FCC, Body–Food Choice Congruence; * loadings > 0.50; ** for a group of items with factor loadings > 0.50.

**Table 3:** Fit measures for the IES-2 models.

Models	Fit Indices					
	*p*	χ^2^/df	TLI	CFI	RMSEA	RMR
Model a	<0.001	6.286	0.892	0.908	0.073	0.105
Model b	<0.001	6.098	0.904	0.919	0.071	0.099
Model c	<0.001	4.613	0.932	0.946	0.060	0.097
Model d	<0.001	3.344	0.958	0.967	0.048	0.066
Model e	<0.001	2.815	0.970	0.976	0.043	0.048

*p*, significance value; χ^2^/df, chi-square fit statistics/degree of freedom; TLI, Tucker–Lewis index; CFI, comparative fix index; RMSEA, root mean square error of approximation; RMR, root mean square residual.

**Table 4:** Measurement invariance of IES-2 across gender.

Models	Fit Measures					Model Comparison			
	χ^2^	df	χ^2^/df	RMSEA	CFI	Comparison	Δ χ^2^	Δdf	*p*
Model 0 *	419.309	190	2.207	0.035	0.969	-	-	-	-
Model 1 **	435.774	202	2.157	0.034	0.968	Model 1 vs. 0	16.465	12	0.171
Model 2 ***	465.199	214	2.174	0.034	0.966	Model 2 vs. 1	29.425	12	0.003
Adj. Model 2 ***	441.084	211	2.090	0.033	0.969	Adj. Model 2 vs. 1	5.31	9	0.806

*p*, significance value; χ^2^/df, chi-square fit statistics/degree of freedom; RMSEA, root mean square error of approximation; CFI, confirmatory fit index; adj., adjusted. * Model 0, configural model (unconstrained); ** Model 1, metric model; *** Model 2, scalar model.

**Table 5:** Mean scores for IES-2 subscales.

Subscales	Total (*N* = 1000) M ± SD	Women (*N* = 1000) M ± SD	Men (*N* = 1000) M ± SD
RHSC	3.47 ± 0.83	3.50 ± 0.84	3.44 ± 0.83
EPR *	3.36 ± 1.02	3.27 ± 1.05	3.46 ± 0.97
B-FCC *	3.31 ± 0.89	3.38 ± 0.90	3.24 ± 0.88
UPE	3.59 ± 0.90	3.62 ± 0.90	3.55 ± 0.89

*N*, number of participants; M, mean; SD, standard deviation; RHSC, Reliance on Hunger and Satiety Cues; EPR, Eating For Physical Rather Than Emotional Reasons; B-FCC, Body–Food Choice Congruence; UPE, Unconditional Permission to Eat; significant at * *p* < 0.05; Mann–Whitney U Test.

**Table 6:** Component loadings for MES items.

MES Statements	Original Factor	Items	Factor 1	Factor 2	Factor 3	Factor 4	Factor 5
I snack when I’m bored. ^a^	UnsEat	24	0.693 *	−0.015	0.293	−0.015	0.108
I eat something without really being aware of it. ^a^	ActAwa	10	0.689 *	0.186	0.170	0.178	−0.174
I eat between meals. ^a^	UnsEat	6	0.688 *	−0.322	0.042	−0.123	0.008
I snack without being aware that I’m eating. ^a^	ActAwa	25	0.651 *	0.182	0.311	0.179	−0.026
I eat automatically without being aware of what I’m eating. ^a^	ActAwa	17	0.646 *	0.200	0.254	0.228	−0.108
I multi-task whilst eating. ^a^	UnsEat	11	0.606 *	−0.089	0.218	0.026	−0.194
I don’t pay attention to what I’m eating because I’m daydreaming, worrying, or distracted. ^a^	ActAwa	5	0.598 *	0.184	0.272	0.242	−0.123
When I get hungry, I can’t think about anything else. ^a^	NonRea	14	0.594 *	−0.081	0.226	0.290	0.375
I eat at my desk or computer. ^a^	UnsEat	18	0.579 *	−0.073	0.076	0.168	−0.202
I become very short-tempered if I need to eat. ^a^	NonRea	21	0.576 *	0.028	0.269	0.289	0.313
Once I’ve decided to eat, I have to eat straight away. ^a^	NonRea	3	0.542 *	−0.207	0.190	0.313	0.239
I notice flavours and textures when I’m eating my food.	Awa	26	0.002	0.797 *	−0.115	0.024	0.047
I notice how my food looks.	Awa	2	−0.006	0.787 *	−0.146	−0.023	−0.011
I notice the smells and aromas of food.	Awa	8	0.000	0.777 *	−0.091	0.053	0.014
I stay aware of my food whilst I’m eating.	Awa	15	0.102	0.757 *	0.030	−0.138	0.116
It’s easy for me to concentrate on what I’m eating.	Awa	23	−0.004	0.630 *	0.006	−0.305	0.053
I wish I could control my eating more easily. ^a^	Acc	7	0.202	−0.174	0.776 *	0.036	0.023
I wish I could control my hunger. ^a^	Acc	1	0.153	−0.164	0.730 *	0.054	−0.059
I criticize myself for the way I eat. ^a^	Acc	27	0.406	0.084	0.643 *	0.174	−0.055
I tell myself I shouldn’t be eating what I’m eating. ^a^	Acc	20	0.432	0.055	0.584 *	0.186	−0.021
I tend to evaluate whether my eating is right or wrong. ^a^	Acc	12	0.189	−0.237	0.565 *	0.257	−0.104
I tell myself I shouldn’t be hungry. ^a^	Acc	13	0.306	−0.006	0.531 *	0.304	0.021
I need to eat like clockwork. ^a^	NonRea	19	0.084	−0.124	0.208	0.783 *	0.100
I have a routine for when I eat. ^a^	Rou	16	0.087	−0.254	0.100	0.721 *	0.075
I eat the same thing on the same day of each week. ^a^	Rou	4	0.398	0.069	0.158	0.553 *	−0.288
I eat the same thing for lunch each day. ^a^	Rou	22	0.438	0.143	0.172	0.521 *	−0.145
I have a routine for what I eat. ^a^	Rou	28	0.354	−0.078	0.190	0.469	−0.323
I can tolerate being hungry for a while.	NonRea	9	−0.192	0.356	−0.144	−0.061	0.639*
*Eigenvalue*			5.392	3.498	3.263	2.762	1.113
*% of Variance explained*			19.256	12.494	11.654	9.863	3.975
Cronbach’s Alpha **			0.885	0.833	0.822	0.733	-

^a^ reverse-coded items; ^b^ original IES-2 number of the statement; Acc, Acceptance; Awa, Awareness; NonRea, Non-reactivity; Rou, Routine; ActAwa, Act with awareness; UnsEat, Unstructured eating; reverse-coded items; * loadings > 0.50; ** for a group of items with factor loadings > 0.50.

**Table 7:** Fit measures for the MES models.

Models	Fit Indices					
	*p*	χ^2^/df	TLI	CFI	RMSEA	RMR
Model a	<0.001	5.732	0.855	0.869	0.069	0.059
Model b	<0.001	5.373	0.877	0.890	0.066	0.055
Model c	<0.001	3.560	0.928	0.938	0.051	0.500
Model d	<0.001	2.926	0.955	0.962	0.044	0.041
Model e	<0.001	3.035	0.957	0.965	0.045	0.043
Model f	<0.001	2.814	0.963	0.970	0.043	0.040

*p*, significance value; χ^2^/df, chi-square fit statistics/degree of freedom; TLI, Tucker–Lewis index; CFI, comparative fix index; RMSEA, root mean square error of approximation; RMR, root mean square residual.

**Table 8:** Measurement invariance of MES across gender.

Models	Fit Measures					Model Comparison			
	χ^2^	df	χ^2^/df	RMSEA	CFI	Comparison	Δ χ^2^	Δdf	*p*
Model 0 *	463.516	222	2.088	0.033	0.964	-	-	-	-
Model 1 **	488.658	236	2.071	0.033	0.963	Model 1 vs. 0	25.142	14	0.033
Adj. Model 1 **	476.969	234	2.038	0.032	0.964	Adj. Model 1 vs. 0	13.452	12	0.337
Model 2 ***	505.238	248	2.037	0.032	0.962	Model 2 vs. Adj. Model 1	28.269	14	0.013
Adj. Model 2 ***	493.017	246	2.004	0.032	0.963	Adj. Model 2 vs. Adj. Model 1	16.048	12	0.189

*p*, significance value; χ^2^/df, chi-square fit statistics/degree of freedom; RMSEA, root mean square error of approximation; CFI, confirmatory fit index; adj., adjusted. * Model 0, configural model (unconstrained); ** Model 1, metric model; *** Model 2, scalar model.

**Table 9:** Mean scores for MES subscales.

Subscales	Total (*N* = 1000) M ± SD	Women (*N* = 1000) M ± SD	Men (*N* = 1000) M ± SD
Act with awareness	3.10 ± 0.63	3.10 ± 0.63	3.11 ± 0.62
Awareness **	2.92 ± 0.73	3.02 ± 0.72	2.82 ± 0.72
Acceptance	2.94 ± 0.69	2.91 ± 0.71	2.98 ± 0.68

*N*, number of participants; M, mean; SD, standard deviation; significant at ** *p* < 0.001; Mann–Whitney U Test.

**Table 10:** Correlations between IES-2 and MES subscales.

Variables	1	2	3	4	5	6	7
1. RHSC	-						
2. APR	−0.048	-					
3. B-FCC	0.461 **	−0.137 **	-				
4. UPE	0.515 **	−0.085 **	0.173 **	-			
5. ActAwa	0.071 *	0.487 **	0.092 **	0.003	-		
6. Awa	0.325 **	0.199 **	0.300 **	0.298 **	0.188 **	-	
7. Acc	0.081 *	0.401 **	−0.109 **	0.148 **	0.627 **	−0.010	-

RHSC, Reliance on Hunger and Satiety Cues; EPR, Eating For Physical Rather Than Emotional Reasons; B-FCC, Body–Food Choice Congruence; UPE, Unconditional Permission to Eat (Intuitive Eating Scale-2); ActAwa, Act with awareness; Awa, Awareness; Acc, Acceptance (Mindful Eating Scale); significant at * *p* < 0.05, ** *p* < 0.01 (Spearman’s correlation).

**Table 11:** Discriminant validity of IES-2 and MES subscales.

Food Groups	Factors (Subscales)						
	ActAwa	Awa	Acc	RHSC	EPR	B-FCC	UPE
	(M ± SD)	(M ± SD)	(M ± SD)	(M ± SD)	(M ± SD)	(M ± SD)	(M ± SD)
Vegetables and fruit							
Low intake ^a^	3.09 ± 0.59	2.74 ± 0.75 ***	3.04 ± 0.66 ***	3.37 ± 0.86	3.45 ± 0.94 *	3.03 ± 0.89 ***	3.54 ± 0.95
High intake ^a^	3.09 ± 0.68	3.12 ± 0.70 ***	2.84 ± 0.72 ***	3.50 ± 0.86	3.25 ± 1.10 *	3.53 ± 0.88 ***	3.58 ± 0.90
Sweets and salty snacks							
Low intake	3.24 ± 0.61 ***	2.93 ± 0.76	2.97 ± 0.68 *	3.37 ± 0.85 *	3.57 ± 0.96 ***	3.41 ± 0.88 **	3.35 ± 0.89 ***
High intake	2.92 ± 0.66 ***	2.88 ± 0.74	2.84 ± 0.73 *	3.51 ± 0.88 *	3.10 ± 1.09 ***	3.18 ± 0.95 **	3.72 ± 0.97 ***

^a^ “low” intake—1st tercile, and “high” intake—3rd tercile; * *p* < 0.05; ** *p* < 0.01; *** *p* < 0.001 (Mann–Whitney test); ActAwa, Act with awareness; Awa, Awareness; Acc, Acceptance (Mindful Eating Scale); RHSC, Reliance on Hunger and Satiety Cues; EPR, Eating For Physical Rather Than Emotional Reasons; B-FCC, Body–Food Choice Congruence; UPE, Unconditional Permission to Eat (Intuitive Eating Scale-2); M, mean; SD, standard deviation.

**Table 12:** Correlations between intuitive eating, mindful eating, and food intake.

Intuitive and Mindful Eating Factors	Group	Food Groups				
		Fresh Vegetables (g)	Processed Vegetables (g)	Fresh Fruit (g)	Sweets (g)	Salty Snacks (g)
ActAwa	Total	−0.054	−0.015	0.060	−0.174 **	−0.253 **
	Women	−0.026	0.026	0.103 *	−0.206 **	−0.249 **
	Men	−0.079	0.010	0.017	−0.138 *	−0.252 **
Awa	Total	0.189 **	0.169 **	0.199 **	0.053	−0.110 **
	Women	0.208 **	0.136 **	0.211 **	−0.002	−0.123 **
	Men	0.129 **	0.169 **	0.130 **	0.098*	−0.091 *
Acc	Total	−0.160 **	−0.053 **	−0.068 *	−0.039	−0.124 **
	Women	−0.149 **	−0.045	−0.022	−0.041	−0.137 **
	Men	−0.163 **	−0.048	−0.104 *	−0.037	−0.112 **
RHSC	Total	0.047	0.056	0.106 **	0.106 **	0.028
	Women	0.058	0.021	0.145 **	0.110 *	0.070
	Men	0.023	0.084	0.055	0.091 *	−0.009
EPR	Total	−0.085	−0.050	−0.050	−0.146 **	−0.186 **
	Women	−0.055	−0.070	−0.020	−0.178 *	−0.204 **
	Men	−0.091 *	−0.004	−0.054	−0.102 *	−0.173 **
B-FCC	Total	0.246 **	0.225 **	0.237 **	−0.112 **	−0.048
	Women	0.273 **	0.229 **	0.224 **	−0.151 **	−0.048
	Men	0.205 **	0.203 **	0.230 **	−0.081	−0.044
UPE	Total	−0.026	0.029	0.066 *	0.255 **	0.051
	Women	−0.060	0.001	0.070	0.308 **	0.123 **
	Men	−0.015	0.040	0.042	0.191 **	−0.021

ActAwa, Act with awareness; Awa, Awareness; Acc, Acceptance (Mindful Eating Scale); RHSC, Reliance on Hunger and Satiety Cues; EPR, Eating For Physical Rather Than Emotional Reasons; B-FCC, Body–Food Choice Congruence; UPE, Unconditional Permission to Eat (Intuitive Eating Scale-2); significant at * *p* < 0.05. ** *p* < 0.01 (Spearman’s correlation).

## Data Availability

The data presented in this study are available on request from the corresponding author.

## Appendix A

The Intuitive Eating Scale-2—Skala Jedzenia Intuicyjnego-2
1. Staram się unikać niektórych produktów o dużej zawartości tłuszczu, węglowodanów lub kalorii.
2. Mam w zwyczaju jeść pod wpływem emocji (np. niepokoju, żalu, smutku), nawet jeśli nie odczuwam akurat głodu.
3. Jeśli mam ochotę na jakiś produkt, nie odmawiam go sobie.
4. Złoszczę się na siebie, kiedy zjem coś niezdrowego.
5. Zdarza mi się jeść, kiedy czuję się samotny/-a, nawet jeśli nie odczuwam akurat głodu.
6. Ufam, że moje ciało da mi znać, kiedy potrzebuje posiłku.
7. Ufam, że moje ciało da mi znać, jakich pokarmów potrzebuje.
8. Ufam, że moje ciało da mi znać, ile mam zjeść.
8. Ufam, że moje ciało da mi znać, ile mam zjeść.
9. Istnieją “zakazane produkty”, na jedzenie których nie pozwalam sobie.
10. Stosuję jedzenie do radzenia sobie z negatywnymi emocjami.
11. Zdarza mi się jeść, kiedy się czymś stresuję, nawet jeśli nie odczuwam głodu.
12. Umiem radzić sobie z negatywnymi emocjami (np. niepokój, smutek) bez sięgania po jedzenie w celu pocieszenia.
13. Kiedy się nudzę, NIE sięgam po jedzenie tylko po to, aby się czymś zająć.
14. Kiedy czuję się samotny/-a, NIE sięgam po jedzenie w celu pocieszenia się.
15. Szukam innych sposobów na radzenie sobie ze stresem i niepokojem niż poprzez jedzenie.
16. Pozwalam sobie na jedzenie produktów, na które w danym momencie mam ochotę.
17. NIE stosuję diet narzucających co, kiedy i/-lub w jakich ilościach mam jeść.
18. Zazwyczaj mam ochotę na spożywanie produktów o wysokiej wartości odżywczej.
19. Spożywam przede wszystkim żywność, dzięki której mój organizm dobrze funkcjonuje.
20. Spożywam przede wszystkim żywność, która daje mojemu ciału energię i kondycję.
21. Polegam na sygnałach głodu, które mówią mi kiedy mam jeść.
22. Polegam na sygnałach sytości, które mówią mi kiedy mam przestać jeść.
23. Ufam, że moje ciało da mi znać, kiedy zakończyć jedzenie.

1—Zdecydowanie się nie zgadzam
2—Nie zgadzam się
3—Nie mam zdania
4—Zgadzam się
5—Zdecydowanie się zgadzam

## Appendix B

The Mindful Eating Scale—Skala Uważnego Jedzenia
1. Chciałbym/-abym umieć kontrolować swój głód.
2. Zwracam uwagę na wygląd żywności.
3. Kiedy zdecyduję, że powinienem/-nam coś zjeść, muszę zjeść posiłek od razu.
4. Jem to samo w poszczególne dni w każdym tygodniu.
5. Nie zwracam uwagi na to, co jem, ponieważ martwię się, pogrążam się w marzeniach lub jestem rozkojarzony/-a.
6. Jem przekąski pomiędzy posiłkami.
7. Chciałbym/-abym, aby kontrolowanie jedzenia było dla mnie łatwiejsze.
8. Zwracam uwagę na zapach żywności.
9. Jestem w stanie przez jakiś czas być głodny/-a.
10. Jem bez zastanawiania się, co jem.
11. Podczas jedzenia wykonuję kilka czynności naraz.
12. Mam tendencję do oceniania tego, czy mój sposób jedzenia jest prawidłowy czy nieprawidłowy.
13. Mówię sobie, że nie powinienem/-nam być głodny/-a.
14. Kiedy jestem głodny/-a, nie mogę myśleć o czymkolwiek innym.
15. W trakcie jedzenia jestem świadomy/-a tego, co spożywam.
16. Pory jedzenia są dla mnie rutyną.
17. Jem w sposób automatyczny, bez zwracania uwagi na to, co akurat spożywam.
18. Jem przy biurku lub komputerze.
19. Muszę jeść o konkretnych porach (jak w zegarku).
20. Mówię sobie, że nie powinienem/-nam jeść produktów, które spożywam.
21. Staję się wybuchowy/-a, kiedy chce mi się jeść.
22. Jem to samo na lunch (posiłek w godzinach południowych) każdego dnia.
23. Z łatwością przychodzi mi koncentrowanie się na tym, co spożywam.
24. Jem przekąski, gdy się nudzę.
25. Sięgam po przekąski NIE będąc świadomym tego, że jem.
26. Zwracam uwagę na smak i teksturę żywności, którą spożywam.
27. Krytykuję siebie za to, w jaki sposób jem.
28. To co jem, jest dla mnie rutyną.

1—Rzadko/nigdy
2—Czasami
3—Często
4—Zazwyczaj/zawsze
